# Proteins linked to type II interferon response in Sjögren’s disease: novel indicators for disease monitoring and predicting treatment response to leflunomide and hydroxychloroquine combination therapy

**DOI:** 10.3389/fimmu.2025.1566377

**Published:** 2025-10-17

**Authors:** Wing-Yi Wong, Helen L. Leavis, Sofie L. M. Blokland, Valentin M. D. Baloche, Joel A. G. van Roon

**Affiliations:** ^1^ Department of Rheumatology and Clinical Immunology, University Medical Center Utrecht, Utrecht, Netherlands; ^2^ Department of Rheumatology, Ziekenhuisgroep Twente, Almelo, Netherlands; ^3^ Center for Translational Immunology, University Medical Center Utrecht, Utrecht, Netherlands

**Keywords:** Sjögren’s disease, leflunomide, hydroxychloroquine, proteomics, clinical trial

## Abstract

**Objective:**

To identify biomarkers and endotypes predictive of treatment response, monitor disease activity, and explore pathways associated with the clinical efficacy of leflunomide and hydroxychloroquine combination therapy (LEF/HCQ) in patients with primary Sjögren’s disease (SjD).

**Methods:**

Serum proteome (Olink Immuno-oncology panel, analyzing 92 proteins) of 29 patients with SjD of the RepurpSS-I study and 8 healthy controls was analyzed at baseline and after 24 weeks of LEF/HCQ. Proteomic changes were correlated to standard and novel clinical endpoints. Transcriptome data of blood mononuclear cells and monocytes were used to assess type I and II IFN scores.

**Results:**

At baseline, 29 proteins were differentially expressed between SjD and HC. LEF/HCQ significantly downregulated 22 out of 27 over-expressed proteins, which was not observed in the placebo-arm. Fourteen baseline proteins and the changes of four of these proteins, CXCL10, CXCL11, TNF, and soluble CD70 concentrations, were correlated with clinical response (|r| 0.40–0.62, p<0.05). Principal Component Analysis revealed an IFN-γ-associated set of coherent proteins. At baseline, using only two proteins, CXCL10 and CXCL11 effectively distinguished patients from healthy controls and responders from non-responders (all p<0.05). Finally, in addition to changes in type I IFN signatures, type II IFN signatures were observed in monocytes that were associated with changes in disease activity.

**Conclusion:**

These data support a significant role for a type II IFN-associated immune response in SjD pathogenesis, which is targeted by LEF/HCQ. Proteins associated with type II IFN-driven immune responses hold potential to monitor disease activity and predict treatment response.

## Introduction

Primary Sjögren’s Disease (SjD) is a chronic systemic autoimmune disease with a prevalence of 0.1-0.5% in the general population that primarily affects women above 50 years old. (1) The disease is characterized by exocrine dysfunction, which leads to symptoms of keratoconjunctivitis sicca and xerostomia. (2) Extra-glandular manifestations are common and about 5-10% of patients develop B-cell lymphoma. (3) These complications are not yet understood and reflect underlying pathogenic processes.

The pathogenesis of SjD is not yet elucidated, but recent advancements have deepened our understanding of the significant role of interferons (IFN) in Sjögren’s disease. Type I interferon has long been recognized as a key driver in SjD. (3) Type I IFN- inducible genes were systemically and locally over-expressed in Sjögren’s patients. (4, 5) Elevated type I IFN levels correlated with the presence of SSA/SSB antibodies. The antibodies link type I IFN to B-cell hyperactivity and immune dysregulation. (6) More recently, type II IFN (IFN-γ) has also been suggested as a potential driver in SjD. (7) A robust type II IFN signature in salivary glands was associated with higher focus scores in salivary gland biopsies and a higher risk of lymphoma. (8) However, the interplay between type I and type II IFN pathways in SjD or how these IFNs contribute to disease activity remains unclear.

There are several critical challenges in SjD. These include disease heterogeneity and the lack of reliable biomarkers for diagnosis and disease monitoring. A valuable framework to monitor disease activity is the EULAR Sjögren’s syndrome disease activity index (ESSDAI). However, the ESSDAI does not capture the entire spectrum of the disease. It does not assess patient-reported outcomes and sicca complaints, which is a key symptom of SjD. (9) To include these, the Sjögren’s Tool for Assessing Response (STAR) and the Composite of Relevant Endpoints for Sjögren’s Syndrome (CRESS) were developed. (10, 11) Yet, both tools are limited in accurately reflecting disease activity in the highly heterogeneous SjD patient group. This limitation emphasizes the need for biomarkers that can reflect systemic and organ-specific disease activity.

To identify biomarkers, proteomics has emerged as a promising approach. Proteomics allow large-scale quantification of proteins, which may provide a basis to discover biomarkers to improve diagnosis, to monitor disease activity, and to predict treatment responses. Proteomics may also provide a detailed view of dysregulations in the immune system underlying SjD, which could improve our understanding of SjD pathogenesis.

Despite the critical need for treatment to prevent clinical complications, no standard treatment is available for SjD. (12) However, there was a breakthrough in 2020 in the RepurpSS-I study: the ESSDAI improved significantly by 4.35 points after 24 weeks of leflunomide and hydroxychloroquine combination therapy (LEF/HCQ). (13) This improvement suggests that LEF/HCQ could robustly inhibit B-cell hyperactivity and significantly alter the disease course in some SjD patients. Yet, the mechanisms underlying this treatment response have not been fully explored.

Our study addresses these critical challenges in SjD by analyzing protein profiles of SjD patients from the RepurpSS-I study. First, we examined whether treatment with LEF/HCQ as compared to placebo can normalize inflammatory protein levels and whether proteomic signatures correlate with clinical response. Second, we tried to identify predictive proteomic biomarkers for clinical endotypes of patients responsive to treatment. Finally, by integrating proteomic data with clinical data, we aimed to provide new insights into the molecular pathways of SjD that are linked to disease activity and clinical response.

## Materials and methods

### Study design and participants

This study involves a comparative analysis of serum protein expression levels between SjD patients and healthy controls (HC), as well as an assessment of LEF/HCQ treatment effects in SjD patients. Samples were collected from SjD patients during the RepurpSS-I clinical trial, and samples from all participants (n = 29) were included in this current study. (13) Of these 29 patients, 21 were randomly allocated to the LEF/HCQ group, and eight received placebo. All patients fulfilled the 2016 ACR-EULAR criteria. At baseline, the ESSDAI score was 10.4 ± 3.9 in the LEF/HCQ group and 9.1 ± 3.4 in the placebo group, anti-SSA antibodies were present in 18/21 (86%) and 7/8 (88%); and anti-SSB antibodies in 13/21 (62%) and 4/8 (50%) in the LEF/HCQ and placebo groups, respectively. The Schirmer test for ocular dryness and unstimulated salivary flow for oral dryness were similar in both groups. None of the patients used concomitant DMARDs or corticosteroids. Additional clinical and demographic details have been reported previously. (13) Eight healthy controls were additionally included, and matched by sex, age, and sample storage duration with the SjD group to ensure group comparability between SjD and HC.

For the assessment of LEF/HCQ treatment effects, SjD patients were divided into groups based on their treatment allocation and clinical response in RepurpSS-I. Responders were defined as patients with an improvement in ESSDAI of at least three points, as previously defined. (9) Accordingly, SjD patients were divided into three groups: placebo (n = 8), LEF/HCQ responders (R; n = 11), and LEF/HCQ non-responders (NR; n = 10). Additional outcome measures included the ESSPRI and newly defined endpoints, STAR and CRESS. The calculation of the STAR and CRESS has been described previously (**Supplementary Material**). (10, 11)

All participants provided informed consent for the RepurpSS-I trial and subsequent sub-analyses. The study was approved by the Medical Ethical Committee of the University Medical Center Utrecht, registered under EudraCT, 2014–003140–12. (13)

### Sample collection and processing

Peripheral blood samples were collected during the RepurpSS-I clinical trial at baseline (T0) and after 24 weeks (T3). For baseline comparisons, samples from all 29 SjD patients in the RepurpSS-I were used. For paired comparisons between T0 and T3, samples from 28 SjD patients were used, due to one placebo drop-out. Serum was separated and stored at -80 °C until analysis. Peripheral blood mononuclear cells (PBMCs) were isolated from whole blood using Ficoll density gradient centrifugation. Monocytes were sorted from PBMCs using MACS cell sorting (human Pan Monocyte Isolation Kit, Miltenyi Biotec) according to manufacturer’s instructions. The purity measured by FACS analysis of the isolated samples was (median [range]) 96% [89-99%], with no significant differences in cell purity between groups. Transcriptomic analysis of whole blood was performed. Cells were lysed in RLTplus buffer (Qiagen) supplemented with 1% β-mercaptoethanol, and total RNA was purified using the AIIPrep Universal Kit (Qiagen) according to manufacturer’s instructions. RNA concentration was assessed using the Qubit RNA kit (Thermo Fisher Scientific), RNA integrity was measured by capillary electrophoresis using the RNA 6000 Nano Kit (Agilent Technologies); all samples had RIN scores > 7.0.

### Protein quantification

Protein levels were quantified using the Olink Immuno-Oncology panel, which measures 92 proteins involved in inflammation and immune regulation. Samples were prepared per the manufacturer’s instructions and analyzed on the Olink platform, measuring all proteins simultaneously. Internal controls and standard curves were included in each run to ensure accuracy and reliability. Data were normalized using the relative protein quantification method provided by Olink, and protein expression levels were reported as Normalized Protein eXpression (NPX) values. NPX values were generated, quality-controlled and adjusted for technical variation by Olink’s proprietary pipeline. This facilitates direct comparison across samples. The NPX value is a quantification unit presented on a log_2_-scale, where a 1-unit difference reflects a doubling of protein concentration (*Olink*. Data normalization and standardization v2.1. 2022). The Olink proteomics data used in this study, including NPX values for all measured proteins, are provided in **Supplementary Table 4**, worksheet ‘Olink’.

### Interferon score assessment

IFN scores were calculated using transcriptomic data from bulk RNA sequencing (detailed procedures in **Supplementary Material**). Only transcriptomic data relevant to the scope of this study were used, specifically for calculating IFN scores. A comprehensive analysis of the full transcriptomic dataset will be reported in a separate publication.

Type I and type II IFN scores were calculated using expression levels of previously described IFN-stimulated genes by Nezos et al., that were over-expressed in salivary glands of patients with SjD. (8, 14) This gene signature was used because it was derived from SjD patients. The genes IFIT1, IFI44, and MX1 were used for type I IFN scores, while GBP1 and CXCL9 were used for type II IFN scores. (14) The mean and standard deviation of each IFN-inducible gene were calculated from the entire study population. The expression levels of these genes were then standardized for each sample using the z-score transformation. The z-score is calculated as z = (x-μ)/σ, where x is the normalized read count, μ the population mean, and σ the population standard deviation. The z-scores for selected IFN-stimulated genes were summed for each subject to provide an IFN type I score (sum of the z-scores for type I IFN-induced genes) and an IFN type II score (sum of the z-scores for type II IFN-induced genes). This methodology was applied to transcriptomic data from PBMCs and monocytes.

To validate the robustness of the experimentally and literature-based IFN scores used in our study, IFN scores were calculated using two alternative gene sets. First, a systemic lupus erythematosus (SLE)-derived signature from Kirou et al. was used, consisting of IFIT1, IFI44, and PRKR for a type I IFN signature, and IRF1, GBP1, SERPING1 for a type II IFN signature. Second, comprehensive and unbiased gene sets were used from the Gene Ontology biological processes (GOBP) “response to type I interferon” (GO:0034340) which included 60 genes, and “response to type II interferon” (GO:0034341) which included 107 genes. (15–18) The gene sets from Nezos et al. used in our primary analyses, along with those by Kirou et al. and GOBP, and transcriptomic data for PBMC and monocytes for the genes included in these signatures are provided in **Supplementary Table 4**.

### Patient and public involvement

Patients or the public were not involved in the design, conduct or reporting of this present study. However, patients contributed to the design of the follow-up study of the RepurpSS-I study, the RepurpSS-II study, which is still ongoing at the time of writing.

### Statistical analysis

All statistical analyses were performed using appropriate methods to ensure the reliability and validity of the findings. Given the small sample sizes and the potential deviation from normality, non-parametric statistical tests were used throughout the study without formal testing for data distribution. The Mann-Whitney *U* test was employed for comparisons between two groups. For paired comparisons between T0 and T3 for each treatment group, the Wilcoxon signed-rank test was used. Benjamini-Hochberg method was used for multiple testing corrections.

Spearman correlation coefficients were calculated for correlation analyses among proteins and between protein expression levels and clinical parameters. The heatmap was generated with hierarchical clustering performed using complete linkage and correlation distance measures.

Principal Component Analysis (PCA) was conducted to reduce the dimensionality of the baseline protein expression data and identify the main sources of variance. The PCA loadings were analyzed to determine the top contributing proteins to the first Principal Component (PC1). Software used for computational analyses can be found in the **Supplementary Material**.

## Results

### Differential protein expression in SjD patients targeted by leflunomide and hydroxychloroquine combination therapy

**Figure 1:**
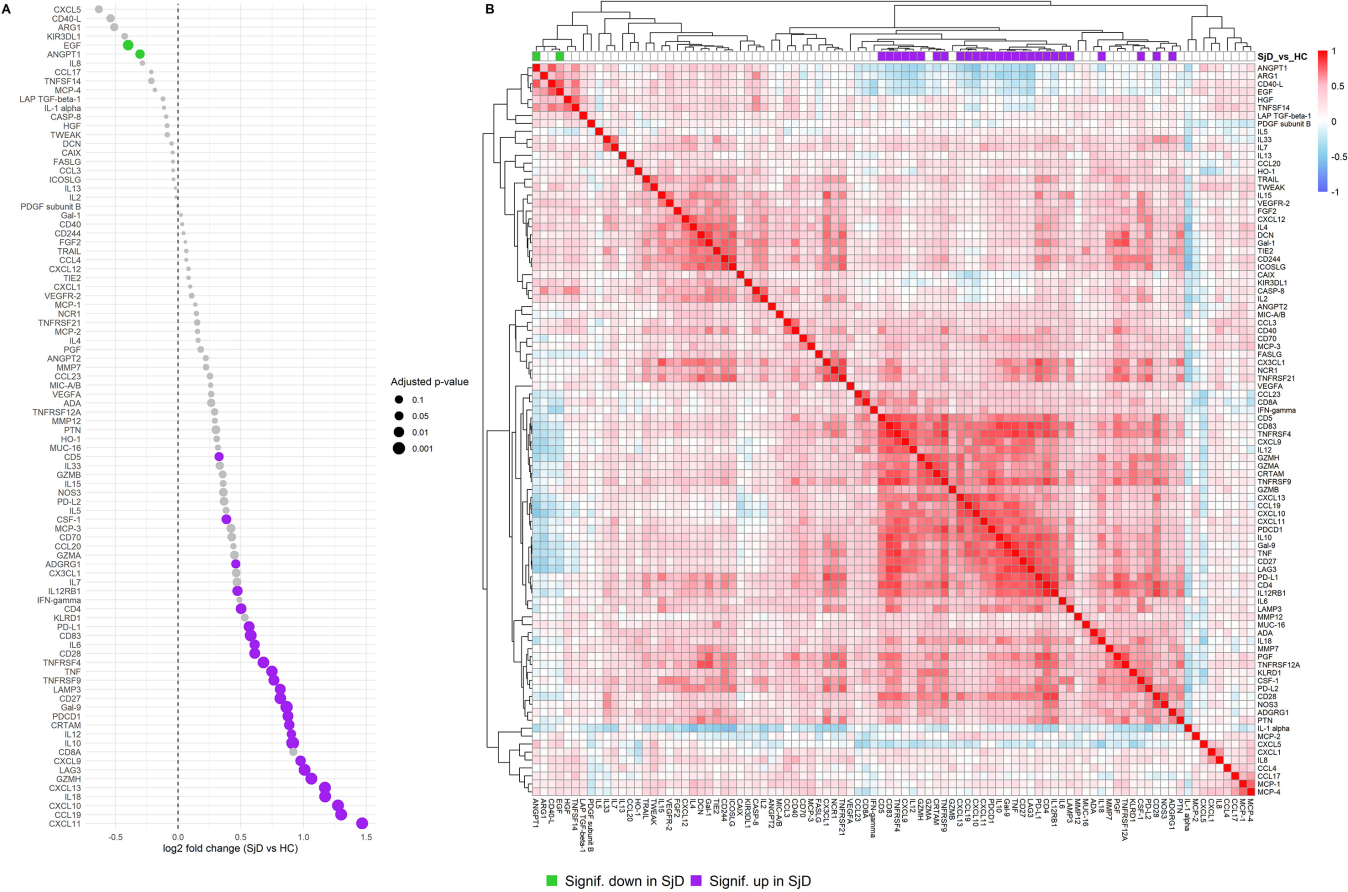
Differential protein expression and protein coherence in SjD patients compared to HC. **(A)** Dot plot showing the log2FC of protein expression levels between SjD patients and HCs at baseline. The x-axis indicates the log2FC compared to HC, and the y-axis lists the proteins by effect size. Each dot represents a protein assay, and dot sizes correspond to the adjusted p-value. **(B)** Heatmap displaying baseline protein correlations among samples from SjD patients and HC groups. Normalized Protein eXpression (NPX) values were used, and Spearman correlations were calculated for each protein pair. Purple: significantly over-expressed proteins in SjD at baseline compared to HC. Lime green: significantly under-expressed proteins in SjD at baseline compared to HC.

First, we compared baseline serum protein profiles of SjD patients (n=29) to HC (n=8) using the Olink Immuno-Oncology panel and identified 29 proteins that were significantly differentially expressed (**Figure 1A**). A detailed list is provided in **Supplementary Table 1**. Among these, 27 proteins were significantly over-expressed in SjD patients. The most significantly over-expressed protein was CXCL11 (log_2_ fold change (log_2_FC) of 1.467, p = 0.0019), followed by CCL19 (log_2_FC = 1.300, p = 0.0019), CXCL10 (log_2_FC = 1.275, p = 0.0019), IL-18 (log_2_FC = 1.17, p = 0.0019), CXCL13 (log_2_FC = 0.976, p = 0.0019), and CXCL9 (log_2_FC = 0.976, p = 0.0118). In contrast, only EGF (log_2_FC = -0.398, p = 0.0118) and ANGPT1 (log_2_FC = -0.305, p = 0.0334) were significantly under-expressed in SjD patients. A correlation heatmap of all proteins revealed coherence of most of the over-expressed proteins (**Figure 1B**).

**Figure 2:**
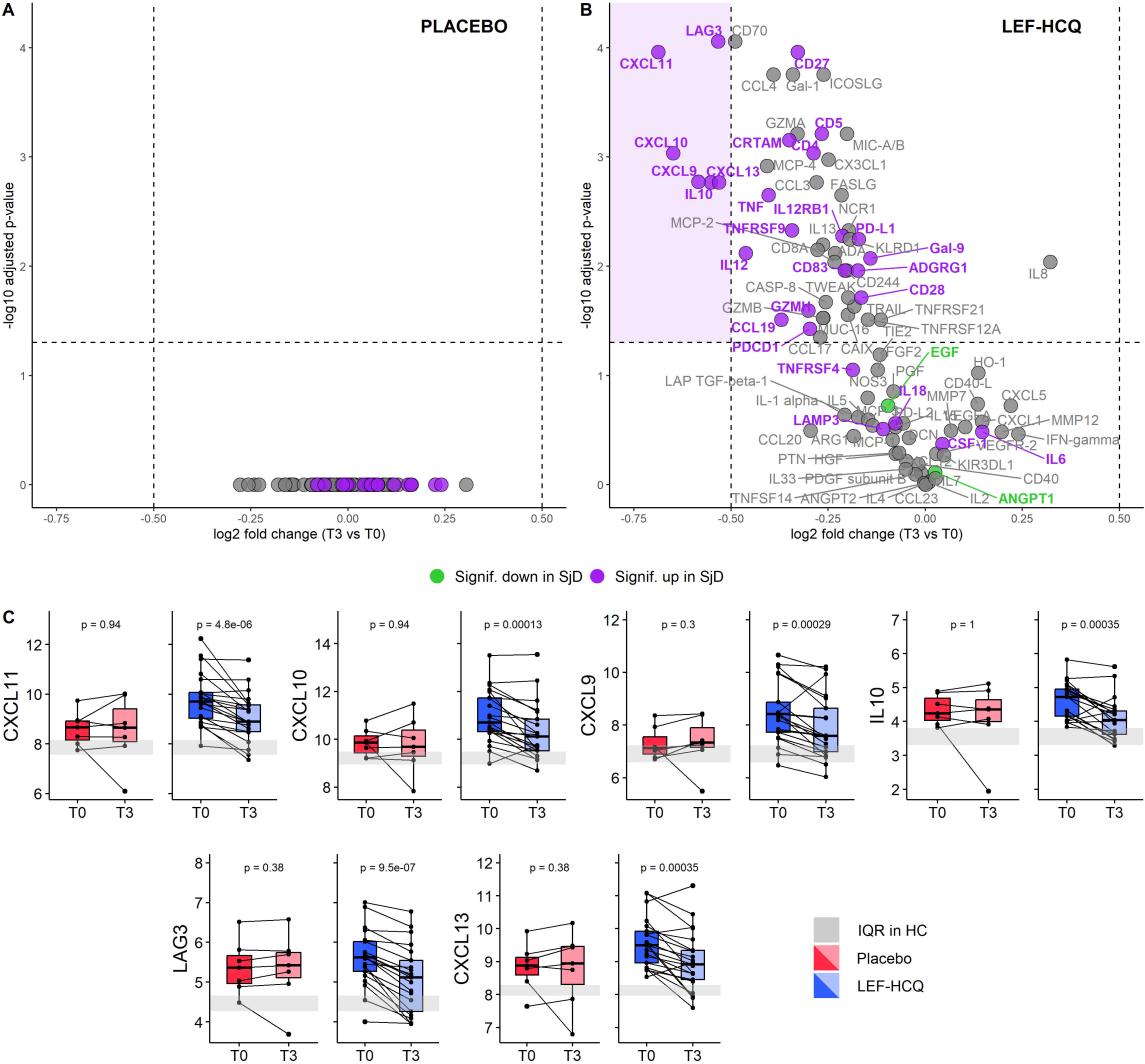
Leflunomide and hydroxychloroquine combination therapy robustly downregulates upregulated inflammatory proteins. **(A, B)** Volcano plots of differential protein expression between T3 and T0 in **(A)** placebo and **(B)** LEF/HCQ group. Each dot represents a protein. The average log2FC is plotted against the -log10 adjusted p-value. The horizontal and vertical dashed lines represent the significance threshold of -log10 adjusted p-value of 0.05 and the log2FC threshold of ± 0.5, respectively. Purple: significantly over-expressed proteins in SjD at baseline compared to HC. Lime green: significantly under-expressed proteins in SjD at baseline compared to HC. **(C)** Paired comparisons of the six most affected proteins at T0 and T3 in the LEF/HCQ group compared to placebo. The grey rectangle represents the interquartile range (IQR) in HC.

Next, we investigated the treatment effect of LEF/HCQ on the complete set of 92 proteins, with special attention to the significant differentially expressed proteins (DEPs) at baseline. Paired analysis of DEPS at T0 and T3 showed no significant changes in the placebo group (**Figure 2A**). In contrast, LEF/HCQ significantly reduced 22 of the 27 over-expressed DEPs with an adjusted p-value <0.05 (**Figure 2B**, all proteins: see **Supplementary Tables 2, 3**). Notably, six of the top over-expressed proteins were robustly downregulated with a log_2_FC of at least -0,5 (CXCL11, CXCL10, CXCL9, IL10, LAG3, and CXCL13; all p <0.05, **Figure 2C**). Protein changes within the LEF/HCQ subgroups, R and NR, are shown in **Supplementary Figure 1**.

### Baseline proteomic analysis identifies a type II IFN-associated protein identifies predictive of response

**Figure 3:**
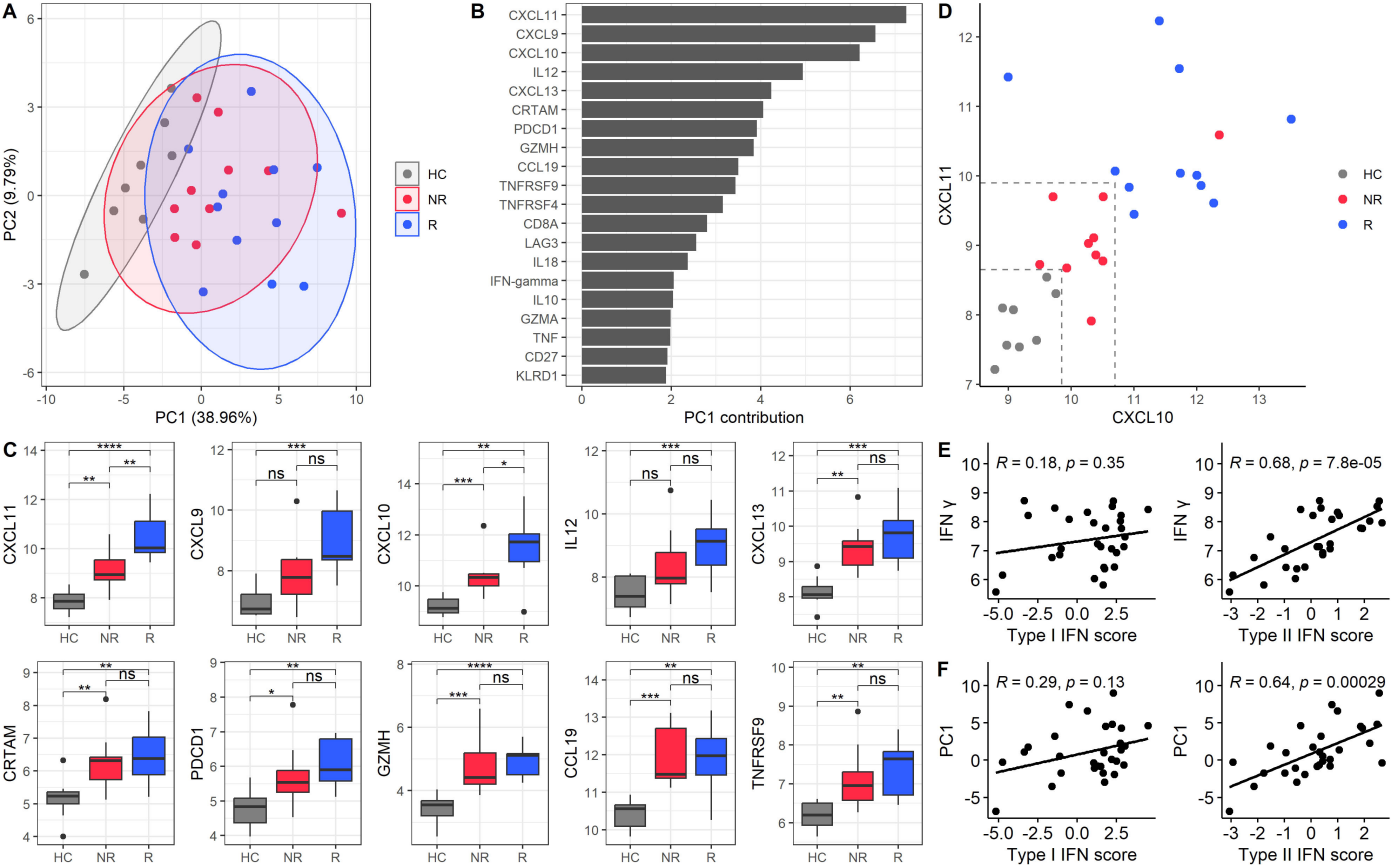
Principal component analysis of baseline protein expression identifies a type II IFN-associated protein component associated with treatment response. **(A)** PCA graphic of baseline samples. PC1 accounts for 38.96%, and PC2 accounts for 9.79% of the variance. The PCA includes all baseline samples, but only HC (grey), NR (red), and R (blue) are shown. Treatment response is based on ESSDAI scores, the current golden standard. Each point represents a sample, and ellipses indicate the 95% confidence interval for each group. **(B)** Bar plot of the top 20 loadings in PC1, ranked by their contribution values. **(C)** Boxplots of expression levels for the top 10 proteins contributing to PC1, comparing HC, NR, and R. **(D)** Scatter plot of CXCL10 and CXCL11 expression levels for HC, NR, and R. Dashed lines indicate arbitrary thresholds that distinguish the different groups at baseline. The cut-off values separating responders form non-responders were 10.7 (CXCL10) and 9.9 (CXCL11). The cut offs separating non-responders from healthy controls were 9.85 (CXCL10) and 8.65 (CXCL11). **(E)** Correlations of IFN-γ protein with type I and type II IFN scores, validating the transcripts in PBMCs. **(F)** Correlations of PC1 with type I and type II IFN scores. Spearman’s correlation coefficients and p-values are provided for panels E and **(F)** *p-value < 0.05, **p-value < 0.01, ***p-value < 0.001, and ****p-value < 0.0001.

Subsequently, we performed an unsupervised analysis to explore the presence of different endotypes based on proteomic profiles. PCA revealed a clear distinction between HC and SjD patients at baseline and between R and NR. Two principal components explained 48.75% of the total variance, with PC1 accounting for 38.96% and PC2 accounting for 9.79% (**Figure 3A**). The proteins contributing most significantly to PC1 included CXCR3 ligands CXCL11, CXCL9, and CXCL10 (**Figure 3B**). We then compared the expression levels of the top ten proteins contributing to PC1 across three groups: HC, NR, and R (**Figure 3C**). For most of these proteins, NR showed expression levels that fell intermediate between those of HC and R. Furthermore, CXCL10 and CXCL11 exhibited significant differences between all three groups. Notably, when combining the expression levels of CXCL10 and CXCL11, HC, NR, and R were effectively distinguished with a notable separation between R and NR (**Figure 3D**). The CXCL10 and CXCL11 expression levels in the treatment groups, defined by newly defined composite scores, STAR and CRESS, can be found in **Supplementary Figure 2**.

Given that the top loadings of PC1 were IFN-γ inducible proteins and IFN-γ was a contributor to the PC1, we investigated whether the entire PC1 was associated with type II IFN activity. To assess this, we calculated type I and II IFN signatures based on RNA transcript levels of PBMCs. The type II IFN score correlated strongly with IFN-γ protein levels (r = 0.68, p = 7.8e-5), confirming the accuracy of the transcriptomic signature for capturing type II IFN activity. In contrast, no significant correlation was observed between IFN-γ and the type I IFN score, which served as a negative control (r = 0.18, p = 0.35) (**Figure 3E**). Building on this validation, we further assessed whether PC1, representing a broader (multidimensional) proteomic profile, was associated with type II IFN activity. We found that PC1 significantly correlated with the type II IFN score (r = 0.64; p = 2.9e-4), whereas no significant correlation was observed between PC1 and type I IFN score (r = 0.29; p = 0.13) (**Figure 3F**). To confirm these findings, we repeated the correlation analyses using two alternative IFN signatures, as described by Kirou et al. and Gene Ontology Biologic Processes atlas (GOBP). (15–18) In both cases, the type II IFN scores were validated based on their correlations with IFN-γ. Results were consistent across all approaches: PC1 correlated with type II IFN score and not with type I IFN score (**Supplementary Figure 5B, C**). These findings indicate the robustness of the IFN signatures for our primary analyses and validate the outcomes. (8)

### Correlation between baseline protein expression and additional clinical outcomes in SjD patients treated with leflunomide/hydroxychloroquine combination therapy

**Figure 4:**
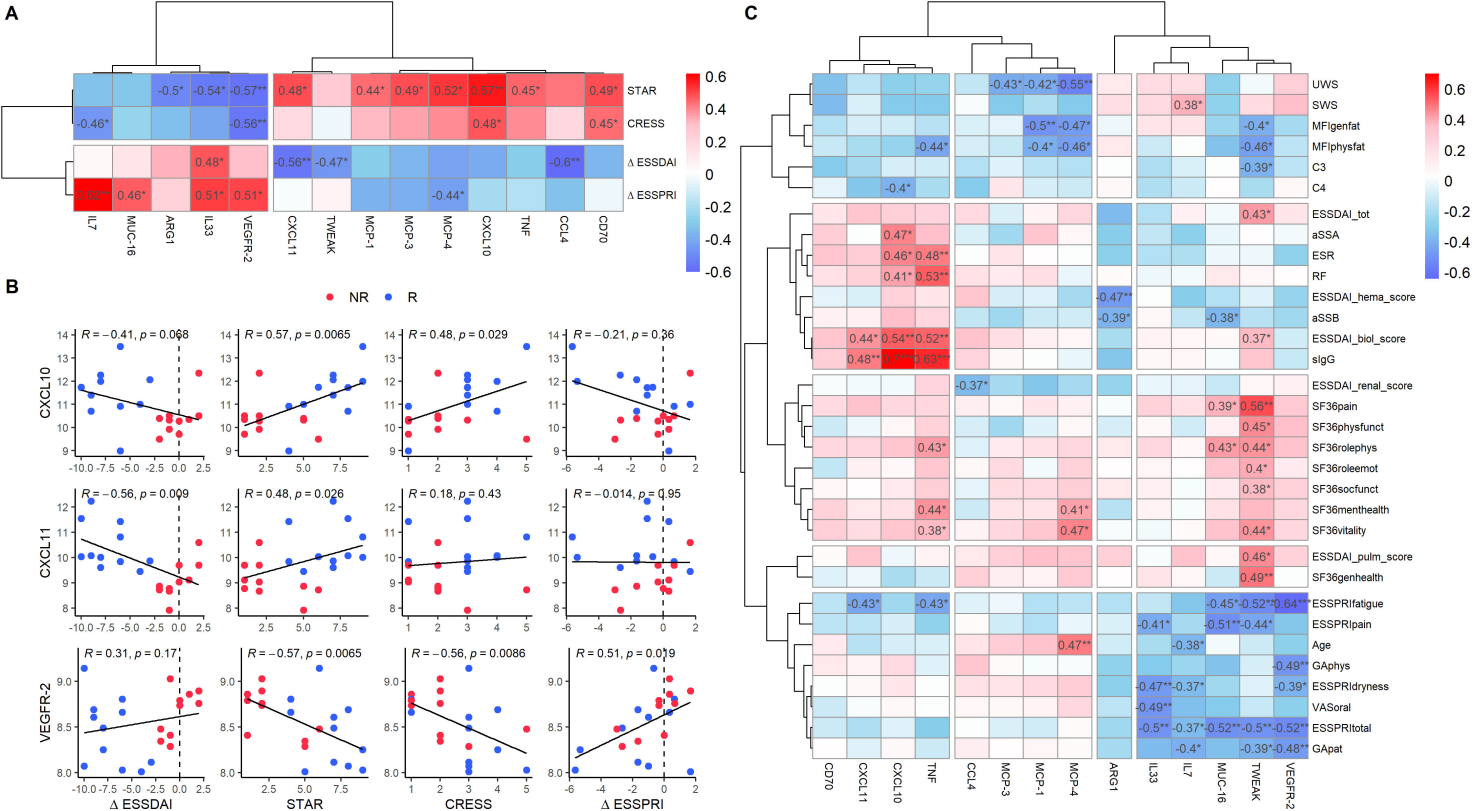
Baseline protein expression shows differential correlation with clinical response parameters in SjD patients. **(A)** Correlation heatmap of baseline protein expression levels and clinical endpoints (STAR, CRESS, ΔESSDAI, ΔESSPRI) in SjD patients treated with LEF/HCQ. Fourteen baseline proteins exhibited significant correlations with clinical endpoints. For this analysis, all proteins from the Olink panel were included, including proteins that showed no statistically significant difference in SjD compared to HC. **(B)** Scatter plots illustrating selected significant correlations between protein expression levels (CXCL10, CXCL11, VEGFR-2) and clinical endpoints. Spearman’s correlation coefficients and p-values are provided. **(C)** Heatmap displaying correlations between the proteins identified in panel A and all clinical parameters at baseline. *p-value < 0.05, **p-value < 0.01, ***p-value < 0.001.

Following our PCA, which revealed baseline differences between R and NR, we further investigated the correlations between baseline protein expression in patients treated with LEF/HCQ and all response measures. In addition to the ΔESSDAI, we included the newly proposed endpoints STAR and CRESS, as they can identify responders based on divergent clinical responses. We also included the ΔESSPRI, the current gold standard for patient-reported outcomes. Fourteen baseline proteins significantly correlated with at least one of the clinical endpoints STAR, CRESS, ΔESSDAI, or ΔESSPRI (**Figure 4A**). Among these proteins, five proteins (IL7, MUC-16, ARG1, IL33, VEGFR-2) were under-expressed in R compared to NR, while nine proteins (CXCL11, TWEAK, MCP-1, MPC-3, MCP-4, CXCL10, TNF, CCL4, CD70) were over-expressed. The serum concentrations of CXCL10 and CXCL11 correlated with the level of improvement of disease activity as captured by (Δ)ESSDAI, STAR, and CRESS scores (**Figure 4B**). Higher levels of CXCL10 and CXCL11 were associated with greater treatment response. Inversely, VEGFR-2 was associated with STAR and CRESS.

Further analysis of the 14 proteins and their correlations with all clinical parameters identified three distinct clusters (**Figure 4C**). Baseline protein over-expression in the first cluster (CD70, CXCL11, CXCL10, TNF) primarily correlated with changes in systemic inflammation. The second cluster (CCL4, MCP-3, MCP-1, MCP-4) correlated with changes in organ-specific parameters. The third cluster proteins (IL33, IL7, MUC-16, TWEAK, VEFR-2) correlated with patient-reported outcomes. A heatmap of the remaining 78 proteins that showed no significant correlations with clinical outcome measures across all clinical parameters is provided in **Supplementary Figure 3**.

### Association of divergent clinical responses with changes in type II IFN-associated protein component and Type II IFN signatures in monocytes in addition to type I IFN signatures

Building on our previous findings, where we identified 14 proteins at baseline that significantly correlated with clinical response, we further explored whether changes in the levels of these proteins during treatment could serve as reliable indicators to monitor disease activity in SjD patients under the treatment of LEF/HCQ. Specifically, we investigated the correlation between the evolution of proteins throughout treatment and clinical endpoints (ΔESSDAI, ΔESSPRI, STAR, and CRESS).

**Figure 5:**
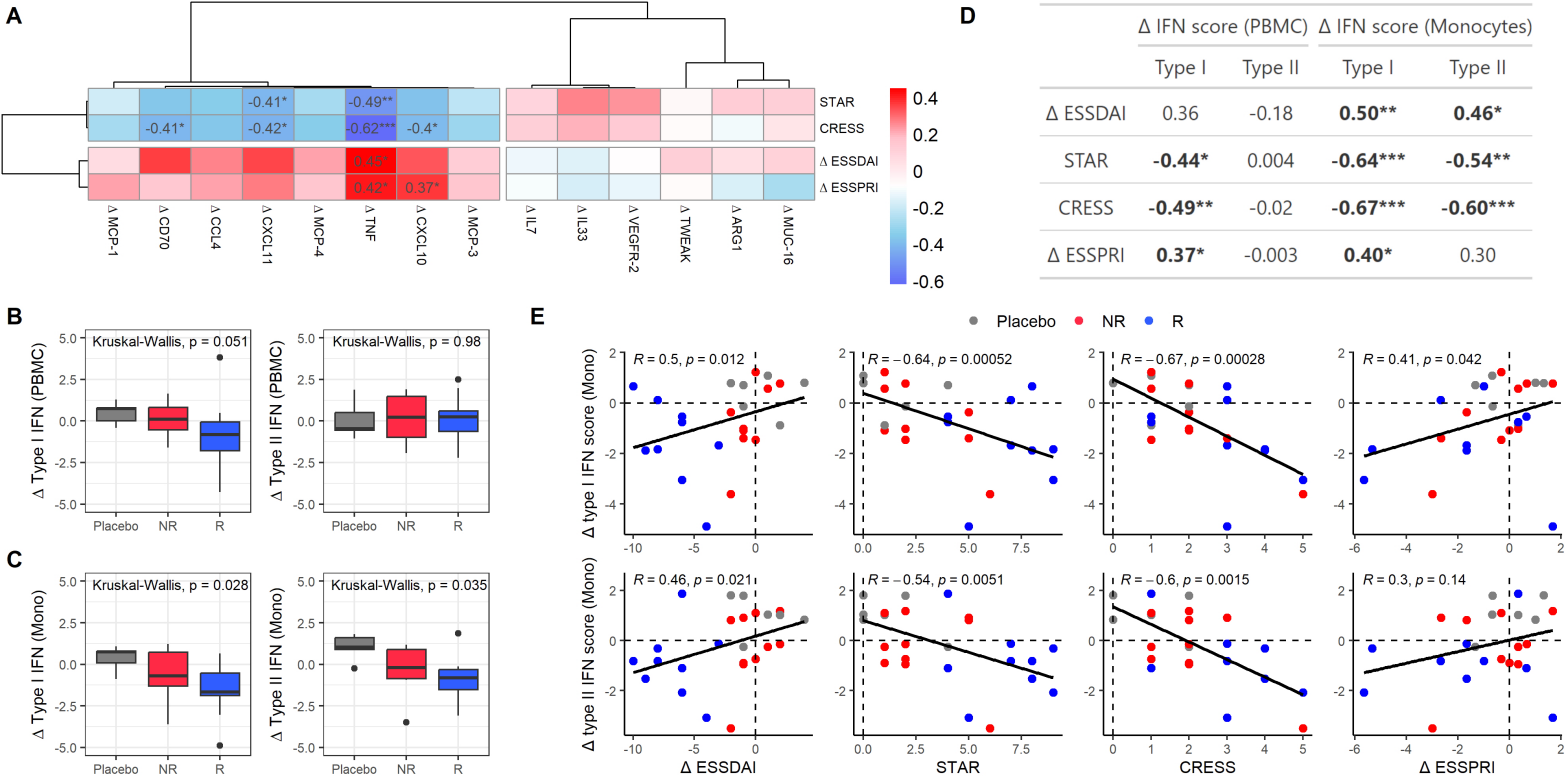
Clinical response is associated with type II IFN-associated proteins and type I and II IFN signatures in monocytes. **(A)** Heatmap showing correlations between protein expression level changes (Δ from T0 to T3) and clinical endpoints in SjD patients treated with either LEF/HCQ or placebo. **(B, C)** Boxplots illustrating changes in type I and II IFN scores from T0 to T3 in **(B)** PBMC and **(C)** monocytes across different treatment groups: Placebo, NR, and R. Statistically significant differences are determined using Kruskal-Wallis tests. **(D)** Table summarizing correlations between changes in type I and type II IFN in PBMCs and monocytes and clinical endpoints (ΔESSDAI, STAR, CRESS, ΔESSPRI). **(E)** Scatter plots showing the relationship between changes in type I and type II IFN scores in monocytes and clinical endpoints. Points are color-coded by treatment group (Placebo, NR, R), and Spearman’s correlation coefficients and p-values are provided. *p-value < 0.05, **p-value < 0.01, ***p-value < 0.001.

Among these 14 proteins, ΔCXCL10, ΔCXCL11, ΔTNF, and ΔCD70 significantly correlated with clinical endpoints (**Figure 5A**). All four proteins correlated with CRESS (r = -0.40, p = 0.035; r = -0.42, p = 0.028; r = -0.62, p = 4.6e-4; r = -0.41, p =0.03, respectively). ΔCXCL10 and ΔTNF correlated with STAR (r = 0.41, p = 0.032; r = 0.49, p = 0.007, respectively). Moreover, ΔTNF was the only protein significantly correlated with ΔESSDAI (r = 0.45, p = 0.015). Finally, both ΔTNF and ΔCXCL10 correlated with ΔESSPRI (r = 0.37, p = 0.027; r = 0.42, p = 0.050). Correlations of all 92 baseline proteins with clinical endpoints are shown in **Supplementary Figure 4**.

Given that CXCL10, CXCL11, and TNF were identified as contributors to the type II IFN-associated PC1 (**Figure 3**), we assessed whether changes in divergent clinical scores were also associated with type I and II IFN gene signatures. Thus, we examined the changes in type I and type II IFN scores over the 24-week treatment period (**Figures 5B, C**). In PBMCs, a trend towards decreased type I IFN scores was observed in responders compared to placebo, which was not observed for non-responders. There were no significant differences in type II IFN scores between these groups.

Monocytes/macrophages are strong producers of CXCL10, CXCL11, and TNF and have been shown to be crucially involved in the immunopathology of SjD, particularly by driving B-cell hyperactivity. The activation of these cells by IFNs is considered critical in the pathogenesis of SjD. (19, 20) Thus, we analyzed IFN signatures specifically in circulating monocytes. Significant differences were observed between placebo, NR, and R for both type I and type II IFN scores in monocytes (**Figure 5C**). We further analyzed the correlations between changes in type I and type II IFN scores and clinical endpoints in monocytes as compared to PBMCs (**Figures 5D, E**). In PBMCs, changes in (delta) type I IFN score correlated significantly with STAR, CRESS, and ΔESSPRI. There were no significant correlations between changes in (delta) type II IFN score and clinical endpoints in PBMCs. In monocytes, delta type I IFN score correlated stronger with ΔESSDAI, STAR, CRESS, and ΔESSPRI than in PBMCs. Additionally, delta type II IFN score in monocytes correlated significantly with ΔESSDAI, STAR, and CRESS. These associations were confirmed when using the alternative IFN signatures defined by Kirou et al. and by GOBP. (15–18) For both type I and type II IFN score changes, correlations with clinical endpoints were stronger in monocytes than in PBMCs (**Supplementary Figures 6B, C**).

## Discussion

In this study, we identified a subset of dysregulated proteins in the serum of SjD patients compared to HC using the Olink Immuno-oncology proteomic panel. LEF/HCQ significantly downregulated most of the upregulated proteins, normalizing proteins to control levels. Type II IFN-associated proteins effectively distinguished SjD patients from HC, and responders from non-responders to LEF/HCQ, indicating a clinical endotype predictive of treatment response. Furthermore, type II IFN-associated proteins were correlated with disease activity and treatment response, highlighting their potential as biomarkers. The treatment response was also associated with type I and type II IFN signatures in monocytes, providing novel insights into the role of monocytic interferon signatures in SjD pathogenesis.

The strongest reductions upon LEF/HCQ treatment were observed in proteins with highest baseline over-expression, including CXCL10 and CXCL11, which also correlated with treatment response. Elevated baseline levels of these chemokines may reflect increased production originating from inflammatory sites, including salivary glands, creating a chemotactic gradient. This is supported by previous reports showing significant over-expression of CXCR3 ligands -CXCL9, CXCL10 and CXCL11- in SjD salivary glands. (7, 21–23) These chemokines, produced by endothelial cells, monocytes, and other cell types, are potent recruiters of CXCR3+ T cells, particularly T_H_1-cells. (24) Subsequently, increased numbers of IFN-γ-producing T_H_1-cells, with IFN-γ being the hallmark T_H_1-cytokine, have been demonstrated in the salivary gland. (7) Consistent with this, decreased circulating frequencies of CXCR3+ T_H_ and IFN-γ-producing T_H_1-cells have been reported, reflecting migration of these cells to inflamed tissues, including the salivary glands. (7, 21) Furthermore, an imbalance in the T_H_1/T_H_2-ratio was linked to markers of SjD disease activity. (7) Finally, IFN-γ is a strong inducer of CXCL10 and CXCL11, contributing to ongoing inflammation in SjD.

The observed decreased CXCL10 and CXCL11 upon LEF/HCQ treatment aligns with reduced IFN-γ activity. This is supported by *in vitro* data showing that *in vivo* concentrations of leflunomide inhibit IFN-γ production, with additive effects when combined with hydroxychloroquine. (25) Taken together, our findings support an IFN-γ-driven CXCL10/CXCl1-mediated T_H_1-cell response that promotes local inflammation and disease activity, which is targeted by LEF/HCQ.

Next to these typical T_H_1-associated cytokines, LEF/HCQ robustly downregulated CXCL13 and IL10, cytokines that activate B-cells. CXCL13 is crucial for B-cells, including B-cell homing and germinal center formation. (26) In SjD, it is associated with disease activity and lymphomagenesis. (27) CXCL13 is also associated with T follicular helper cells (Tfh). (26) Corroborating this, CXCL13 mRNA expression strongly correlated with Tfh cell numbers in SjD salivary glands. (28) Also, downregulation of CXCL13 implicates inhibition of lymphoid neogenesis, strongly affecting B-cell hyperactivity. IL10 is another cytokine that enhances B-cell activity and is associated with T_H_1-driven autoimmunity in SjD. (29) Although speculative, based on inhibition of CXCL13 and CXCR3 ligands a potential target of LEF/HCQ may be CXCR3/CXCR5+ Tfh cells, which were shown to be vigorous producers of IFN-γ, IL-21 and IL10. (30) The strong downregulation of CXCR3 and CXCR5 ligands and IL10 by LEF/HCQ may certainly inhibit Tfh and B-cell hyperactivity, processes driven by type II IFN signaling in SjD.

Interestingly, CXCL10, CXCL11, and CXCL13 were among the top loadings in the type II IFN-associated Principal Component 1. This suggests a connection between CXCL13 and IFN-γ. Experimental models previously showed that IFN-γ excess induced pathogenic accumulation of Tfh and germinal centers. (31) Additionally, IFN-γ receptor signaling in B-cells is central to germinal center formation and autoimmunity. (28, 32) Furthermore, CXCL10 and CXCL11 alone were sufficient to effectively distinguish responders from non-responders to LEF/HCQ, based on ESSDAI, the current golden standard. The unsupervised nature of this analysis further reinforces the distinctive role of CXCL10 and CXCL11 and their potential as biomarkers. With validation in larger studies, this could offer a powerful tool for a personalized treatment approach, enabling clinicians to identify patients that could benefit from LEF/HCQ.

Expanding on this, an additional 14 baseline proteins were identified with the potential to predict a more diverse treatment response, considering recently defined clinical endpoints and patient-reported outcomes. Among these proteins, CXCL10, CXCL11, TNF, and CD70 were identified with the potential to monitor disease activity, as their baseline concentrations correlated with treatment response. TNF contributed to the type II IFN-associated component, which aligns with the described co-expression of IFN-γ and TNF by T_H_1-cells. (33) TNF synergizes with IFN-γ to promote inflammation, including upregulating CXCL10 and CXCL11. (34) IFN-γ also polarizes monocytes/macrophages into pro-inflammatory M1 macrophages, which can further enhance T_H_1 activity associated with IFN-γ production. (35) Additionally, CD70 is implicated in the loss of regulatory T-cell function and facilitates IFN-γ production and T-cell activation. Soluble CD70 and CD27 can result in CD70/CD27 co-stimulation, leading to cytokine production by CD4+ and CD8+ T-cells, such as IFN-γ and CXCL11. (33, 36) (31) Importantly, CD27 was also part of the type II IFN-associated component. Hence, our data suggest that several type II IFN-associated proteins hold the potential to monitor disease activity and predict diverse treatment responses. These proteins are part of a complex type II IFN-associated immune response in SjD.

The exact contribution of type II IFN-associated immune responses in SjD is largely unclear. In our study, the type II IFN-associated protein principal component correlated with IFN-γ protein concentrations and type II IFN-induced signature. Importantly, the protein dynamics of this component did not correlate with type I IFN signature. While proteins like CXCL10 can be induced by type I IFNs, our data suggest that the first principal component is preferentially driven by type II IFNs. While dimensionality reduction through PCA helps capture complex, high-dimensional patterns, the association of PC1 with IFN-γ does not imply that each contributing protein is independently or exclusively induced by this cytokine. Furthermore, we showed that the type II IFN signature in monocytes correlated with systemic disease activity, complementing our findings at protein expression level. The type II IFN signature typically emerges in later stages of the immune response, as activated lymphocytes predominantly express it. (37) Higher type II IFN signature may indicate a sustained immune response from continuous lymphocyte activation. Our data indicate a significant contribution of type II IFN-driven immunopathology, complementing the well-established role of type I IFNs in SjD.

Type I IFNs have long been acknowledged as a key driver in SjD, contributing to autoimmunity and B-cell hyperactivity. (38) Recently, type I IFN signature was shown to be downregulated by hydroxychloroquine. (39) This downregulation did not correlate with systemic disease activity. (39) To our knowledge, successful targeting of type I IFN signature impacting systemic disease activity has not been previously demonstrated in SjD. In line with this, we also did not observe a correlation between type I IFN signatures in PBMCs and systemic disease activity (as measured by ESSDAI). However, we did establish a distinct correlation between changes in type I IFN signature specifically in monocytes and changes in systemic disease activity following LEF/HCQ. This difference between PBMC and monocytes may arise from monocytes constituting only 10% of PBMC, attenuating IFN signatures, and treatment likely affecting only a subset of monocytes. Monocytes may be more sensitive to LEF/HCQ, particularly through mechanisms targeting IFN signaling pathways. Reinforcing this, it has been previously demonstrated that the serum of SjD patients, but not HCs, can induce IFN gene signatures in healthy monocytes. This expression could be partially inhibited by IFNAR1 blockade. (20) This underscores that monocytes in SjD are highly sensitive to type I IFN signaling induced by IFNs. (40)

Although numerous over-expressed proteins were reduced in most patients, the disease inhibition was not complete or even absent in some patients. The sustained disease activity may involve other immune pathways, such as pathways that stimulate IFN-γ production. One potential contributor is IL-18. IL-18 is over-expressed in SjD and was not affected by LEF/HCQ. (41) IL-18 enhances IFN-γ production by T-cells and NK-cells, potentially sustaining disease activity, even in responders. Similarly, IL-6 was not affected by LEF/HCQ. Given the clear disease inhibition by LEF/HCQ this suggests an inferior role of IL-6, which is supported by the previously reported lack of response of IL-6R blockade in SjD. (42) It remains to be shown whether optimized treatment targeting resistant pro-inflammatory proteins, such as IL-18 and IL-6, can be achieved through combination therapy. These examples highlight the importance of future research to understand inflammatory pathways in SjD pathogenesis better.

While our study offers important and novel insights, it also has limitations. First, our cohort consisted of patients with moderate to high disease activity, which may limit the generalizability of our findings. Second, although placebo-controlled, the data derive from a relatively small sample size. The study was sufficiently powered to detect only larger effects, while smaller effects necessitate a larger study. Notably, several inflammatory proteins showed a trend toward normalization, which might have reached statistical significance in a bigger sample size. Third, the Olink Immuno-Oncology panel was a selection of proteins and may introduce a bias. Analyses using a wider range of proteins, such as Olink Explore, could help to reveal a more unbiased representation of SjD immunopathology.

Two confirmatory randomized, double-blind, placebo-controlled trials of LEF/HCQ in SjD are currently ongoing. The RepurpSS-II study evaluated LEF/HCQ in SjD patients with moderate to high disease activity and enrolled 46 patients from centers in the Netherlands. The second, the European NECESSITY study, evaluates the effect of LEF/HCQ in two cohorts: one with moderate to high disease activity and one with low disease activity, with a total target of 100 patients across both cohorts, and 50 patients in the placebo arm. Results from RepurpSS-II are expected by the end of 2025 and from NECESSITY in 2026, allowing clinical and molecular validation.

In conclusion, this study is the only RCT demonstrating improvement in systemic disease activity without allowing concomitant DMARD use, which makes it uniquely valuable for evaluating treatment effects and disease-specific changes. Our findings emphasize the role and targeting of a T_H_1-mediated, type II IFN-driven immune response in SjD pathogenesis, as well as the potential of type II IFN-associated proteins and monocyte-specific IFN scores as biomarkers in Sjögren’s disease. Our findings suggest that these biomarkers could help to broadly monitor immunomodulatory effects in SjD patients and predict treatment response, which may not be limited to LEF/HCQ treatment. With validation, these biomarkers could contribute to more personalized treatment, optimizing treatment strategies and improving patient outcomes while minimizing unnecessary treatments.

## Data Availability

The original contributions presented in the study are included in the article/**Supplementary Material**, further inquiries can be directed to the corresponding author/s.

## Supplementary material

Supplementary Table 1 Proteomics, baseline SjD vs HC.

Supplementary Table 2 Proteomics, T0 vs T3 in placebo.

Supplementary Table 3 Proteomics, T0 vs T3 in LEF-HCQ.

Supplementary Table 4 Annotations, proteomics and RNAseq data.
