# Supplementary material for: Proteins linked to type II interferon response in Sjögren’s disease: novel indicators for disease monitoring and predicting treatment response to leflunomide and hydroxychloroquine combination therapy
Source: Front Immunol. 2025 Oct 17;16:1566377. doi: 10.3389/fimmu.2025.1566377 (PMC12575357; doi:10.3389/fimmu.2025.1566377)
Supplement: Supplementary file 5 [file Table5.docx]

Supplementary Figure Legends

**Supplemental Figure 1. Leflunomide/hydroxychloroquine therapy robustly downregulates upregulated inflammatory proteins in both non-responders and responders. (A-B)** Volcano plots of differential protein expression between T3 and T0 in **(A)** placebo, **(B)** non-responders and **(C)** responders to LEF/HCQ. The average log2 fold change is plotted against the -log10 adjusted p-value. Horizontal and vertical dashed line represents significance threshold of -log10 adjusted p-value of 0.05 and the log2 fold change threshold of ± 0.5 respectively. Purple: significantly overe-xpressed proteins in SjD at baseline compared to HC. Lime green: significantly under-expressed proteins in SjD at baseline compared to HC. **(C)** Paired comparisons of the four most affected proteins at T3 for placebo, non-responders and responders. The grey rectangle represents the interquartile range (IQR) in HC. P-values are indicated.

**Supplemental figure 2.** **Associations between the expression of CXCL10 and CXCL11 and responders and non-responders as captured by the ESSDAI, STAR, and CRESS.** Scatter plots of CXCL10 and CXCL11 expression levels, with points coloured by treatment response (grey= HC, red= NR, blue= R) as defined by clinical endpoints: **A)** The ESSDAI, **B)** STAR and **C)** CRESS. Dashed lines indicate arbitrary thresholds that distinguish the different groups at baseline.

**Supplemental figure 3.** **Correlation heatmap of all baseline proteins with clinical parameters.**

**Supplemental figure 4. Heatmap of correlations between changes in protein levels and clinical parameters.**

**Supplemental Figure 5. Correlation of PC1 with type I and type II interferon (IFN) scores calculated using three different literature-based IFN-related gene sets.** In each approach, the correlation of IFN scores with IFN-γ was assessed to validate the performance of these different. The correlation of IFN-γ type I IFN scores served as negative control. Additionally, the correlation of IFN scores with PC1 was assessed. In this analysis the robustness of the IFN scores used in the main analysis (A, gene sets by Nezos et al.) were compared with alternative IFN scores (B, based on gene sets by Kirou et al.) and IFN scores based on curated gene sets by Gene Ontology biological processes (panel C, “response to type I interferon” (GO:0034340) and “response to type II interferon” (GO:0034341).

**Supplemental Figure 6.** **Correlation of changes in IFN scores with changes in clinical endpoints, comparing PBMCs and monocytes, using three different IFN gene sets.** For each gene set, correlations are shown between the change in IFN score from T0 to T3 with clinical endpoints (delta/change in ESSDAI and ESSPRI and STAR and CRESS response points). Across all three gene sets, correlations were consistently stronger in monocytes than in PBMCs, supporting the robustness of the IFN signature in monocytes in SjD patients. (A) IFN scores based on gene sets by Nezos et al. used in this paper, (B) IFN scores based on gene sets Kirou et al. and (C) IFN scores based on gene sets Gene Ontology biological processes (“response to type I interferon” (GO:0034340) and “response to type II interferon”, GO:0034341).
