# Supplementary material for: Proteins linked to type II interferon response in Sjögren’s disease: novel indicators for disease monitoring and predicting treatment response to leflunomide and hydroxychloroquine combination therapy
Source: Front Immunol. 2025 Oct 17;16:1566377. doi: 10.3389/fimmu.2025.1566377 (PMC12575357; doi:10.3389/fimmu.2025.1566377)
Supplement: Supplementary file 6 [file Table6.docx]

Supplementary Materials and Methods

**Software and packages**

Data analysis and visualisation were performed using Rstudio v4.3.1 and the following packages: OlinkAnalyze 3.5.1, readODS 2.1.0, tidyverse 2.0.0, Hmisc 5.1-1, pheatmap 1.0.12, cowplot 1.1.1, ggplotify 0.1.2, ggrepel 0.9.3, ggpubr 0.6.0, FactoMineR 2.9, rstatix 0.7.2, gt 0.10.1.

**RNA sequencing**

RNA sequencing was performed at Genewitz using an Illumina HiSeq 4000 sequencer (Illumina) following the standard manufacturer’s protocols. A Strand-specific RNA library preparation with poly-A selection was performed. Approximately 20 million 150 base pairs paired-end reads were generated for each sample. RNA sequencing data analysis was performed as previously described. Briefly, quality control of the reads was assessed with the FastQC tool, and all samples passed the quality check. Next, reads were aligned to the human genome GRCh38 using the STAR aligner. The Python package HTSeq was used to calculate the read counts for each annotated gene. Raw read counts were filtered to retain genes with ≥10 reads in at least 90% of the samples per dataset. Variance-stabilising transformation was applied to obtain normalised gene counts (variance-stabilised data), which were used for subsequent analyses. Normalization was performed on the full set of transcripts and the genes included in the predefined signatures are shown in Supplementary Table 4 (Excel file, worksheet ‘PBMC’ and ‘monocytes’).

**STAR and CRESS scoring**

The Sjögren’s Tool for Assessing Response (STAR) contains five domains and patients are considered responders when they have at least five points.(1) The scoring is as follows:

| **Domains** | **Points** | **Definition of Response** |
| --- | --- | --- |
| **1. Systemic activity** | 3 | Decrease of ≥3 in clinESSDAI. |
| **2. Patient-reported outcome** | 3 | Decrease of ≥1 point or ≥15% in ESSPRI. |
| **3. Lacrimal gland function** | 1 | Schirmer’s test: |
|  |  | - If abnormal at baseline: increase ≥5 mm from baseline. |
|  |  | - If normal at baseline: no change to abnormal. |
|  |  | Ocular staining score: |
|  |  | - If abnormal at baseline: decrease of ≥2 points from baseline. |
|  |  | - If normal at baseline: no change to abnormal. |
| **4. Salivary gland function** | 1 | Unstimulated whole salivary flow: |
|  |  | - If score >0 at baseline: increase of ≥25% from baseline. |
|  |  | - If score = 0 at baseline: any increase from baseline. |
|  |  | Ultrasound: |
|  |  | - Decrease of ≥25% in total Hocevar score from baseline. |
| **5. Biological** | 1 | **Serum IgG level**: decrease of ≥10%. |
|  |  | **RF level**: decrease of ≥25%. |
| **Candidate STAR responder** | ≥5 points |  |

The Composite of Relevant Endpoints for Sjögren’s Syndrome (CRESS) contains five domains and patients are considered responders when they respond in at least three out of five domains.(2) The scoring is as follows:

| **Measurement** | **Definition of Response** |
| --- | --- |
| **Systemic disease activity** | ClinESSDAI: score of <5 points, indicating low disease activity |
| **Patient-reported symptoms** | ESSPRI: decrease of ≥1 point or ≥15% from baseline |
| **Tear gland*** | Schirmer’s test and OSS: |
|  | - If abnormal Schirmer (5 mm or less) at baseline: increase of ≥5 mm in Schirmer from baseline; |
|  | - If abnormal OSS (≥3 points) at baseline: decrease of ≥2 points in OSS from baseline; |
|  | - If both Schirmer and OSS normal score at baseline (Schirmer >5 mm and OSS of <3 points): no change to abnormal in Schirmer and OSS. |
| **Salivary gland** | Unstimulated whole saliva (UWS) and salivary gland ultrasound (SGUS): |
|  | - UWS: Increase of ≥25%, or if score is 0 mL/min at baseline, any increase from baseline; |
|  | - SGUS: Decrease of ≥25% in total Hocevar score from baseline. |
| **Serological** | Rheumatoid factor and IgG: |
|  | - Rheumatoid factor: Decrease of ≥25% from baseline; |
|  | - IgG: Decrease of ≥10% from baseline. |
| **CRESS responder** | Responder on at least 3 of 5 items |

**References**

1. Seror R, Baron G, Camus M, Cornec D, Perrodeau E, Bowman SJ, et al. Development and preliminary validation of the Sjögren’s Tool for Assessing Response (STAR): a consensual composite score for assessing treatment effect in primary Sjögren’s syndrome. Ann Rheum Dis. 2022 Jul 1;81(7):979–89.

2. Arends S, de Wolff L, van Nimwegen JF, Verstappen GMPJ, Vehof J, Bombardieri M, et al. Composite of Relevant Endpoints for Sjögren’s Syndrome (CRESS): development and validation of a novel outcome measure. Lancet Rheumatol. 2021 Aug 1;3(8):e553–62.
