# Supplementary figures and images for: Proteins linked to type II interferon response in Sjögren’s disease: novel indicators for disease monitoring and predicting treatment response to leflunomide and hydroxychloroquine combination therapy
Source: Front Immunol. 2025 Oct 17;16:1566377. doi: 10.3389/fimmu.2025.1566377 (PMC12575357; doi:10.3389/fimmu.2025.1566377)

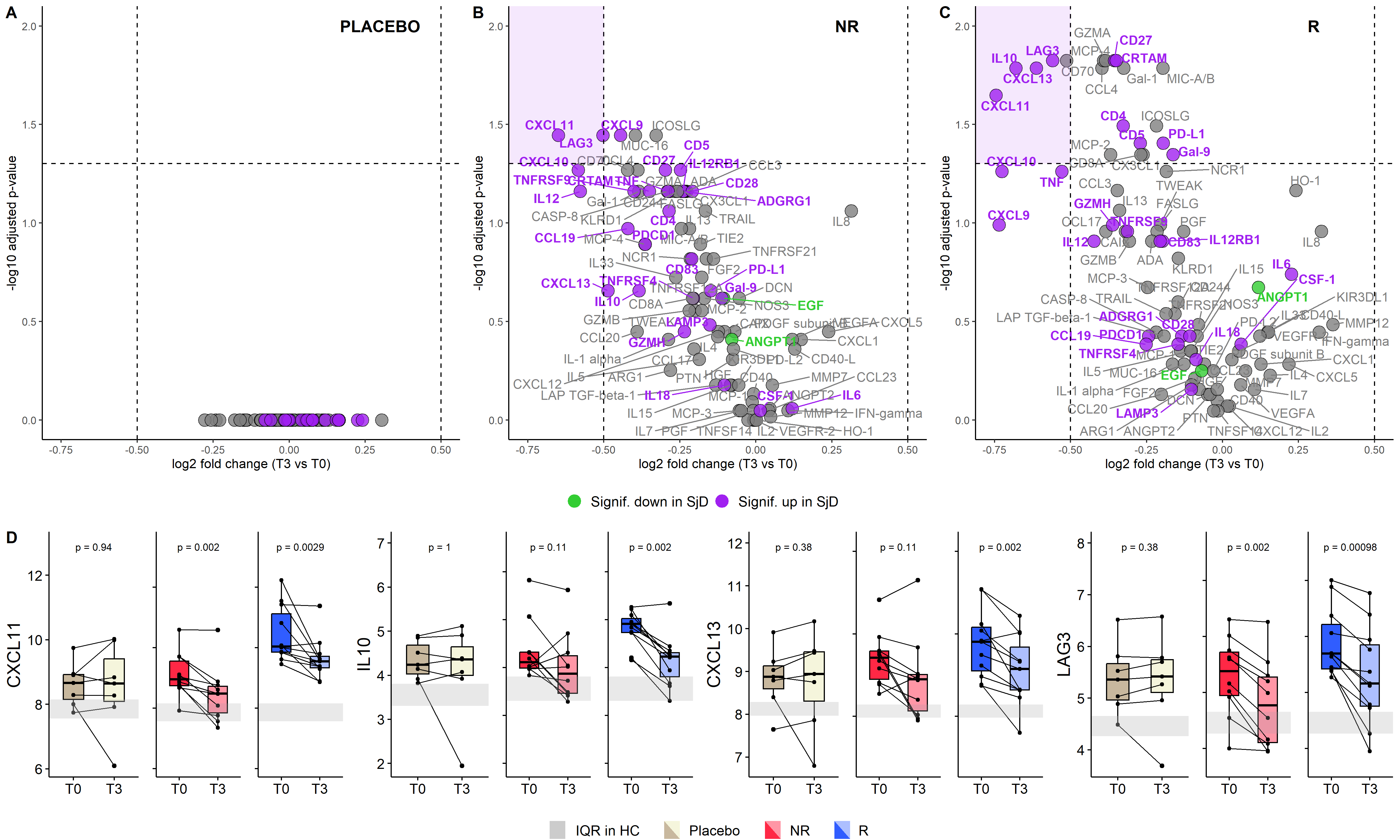

Supplement: Supplementary file 7 [file Image1.png]

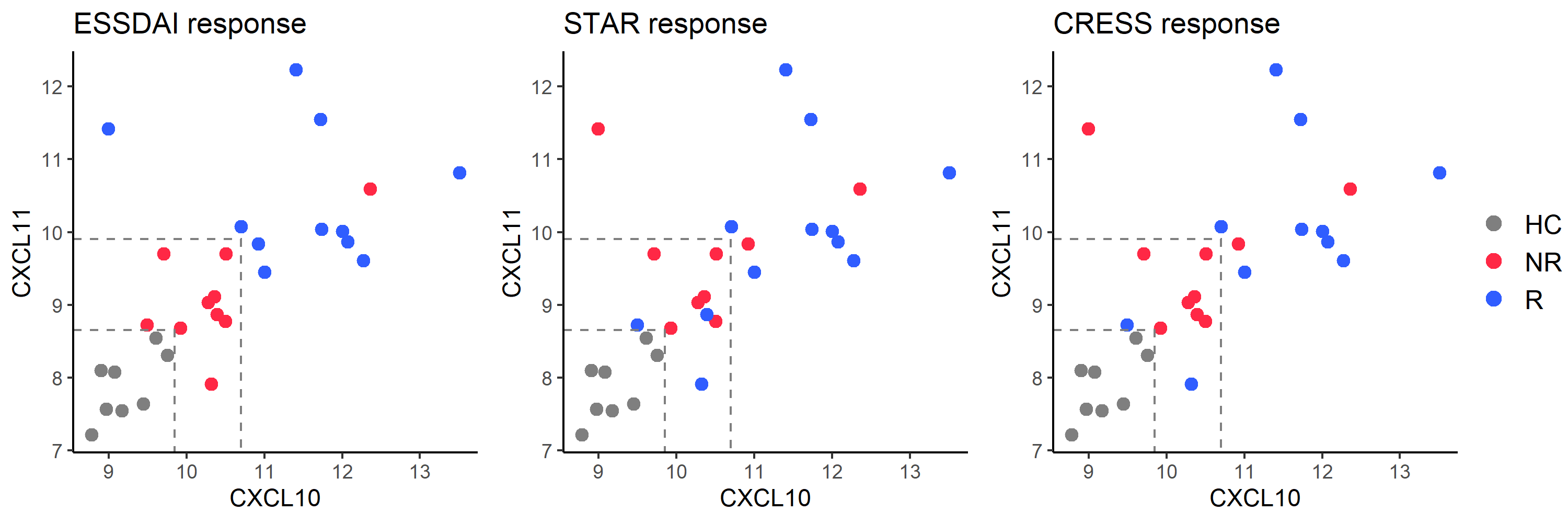

Supplement: Supplementary file 8 [file Image2.png]

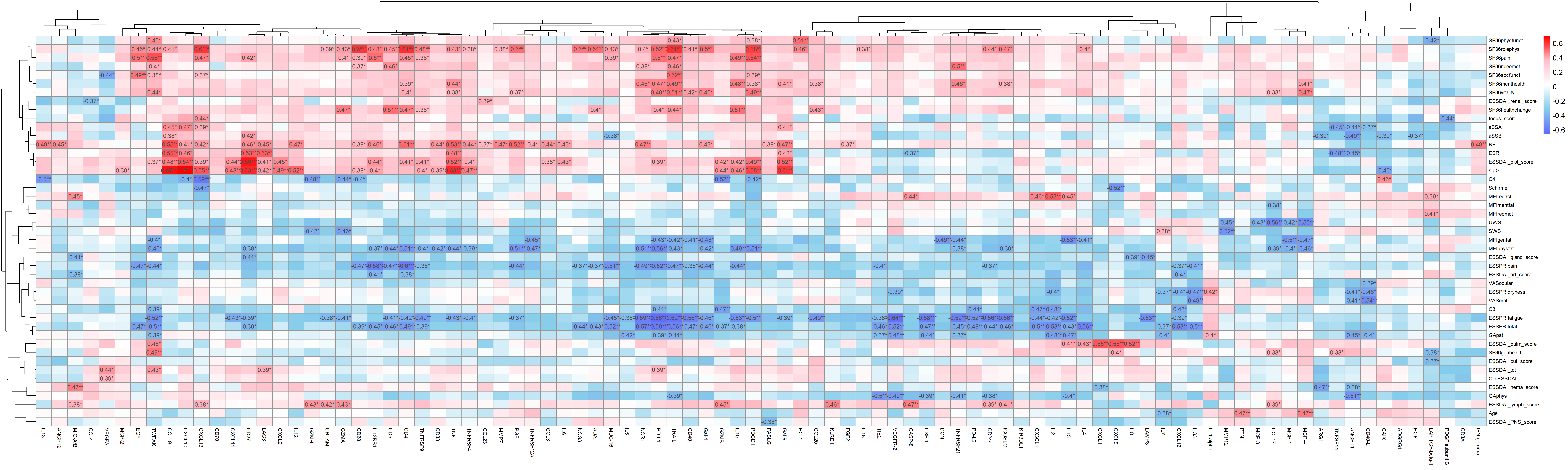

Supplement: Supplementary file 9 [file Image3.png]

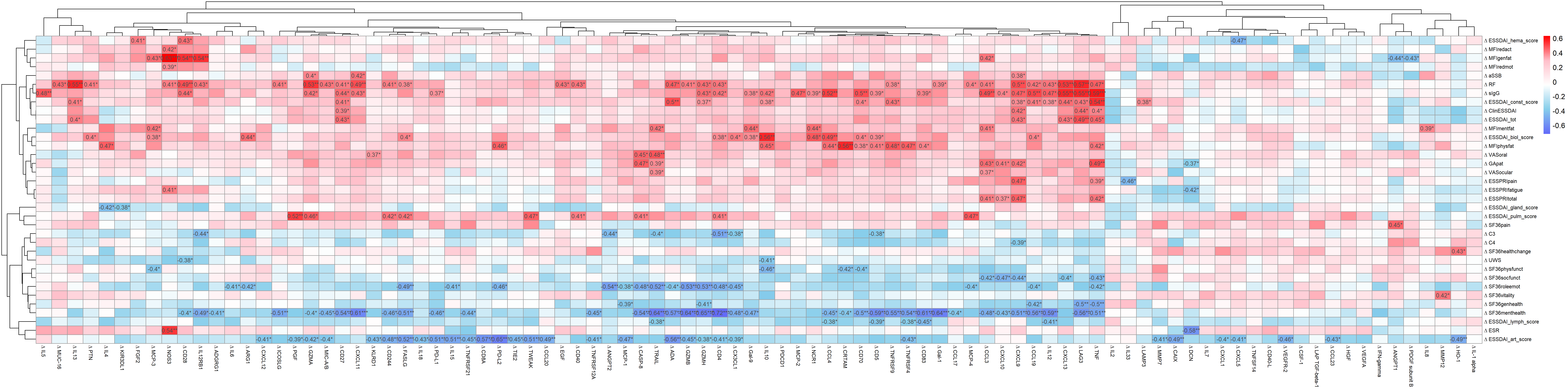

Supplement: Supplementary file 10 [file Image4.png]

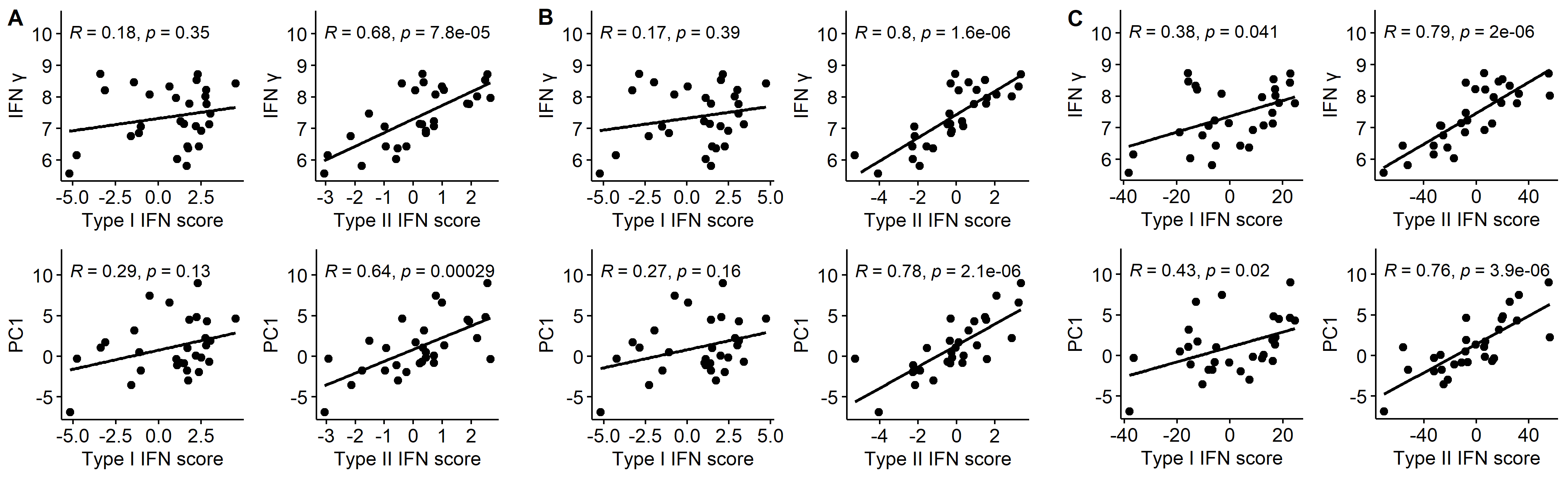

Supplement: Supplementary file 11 [file Image5.png]

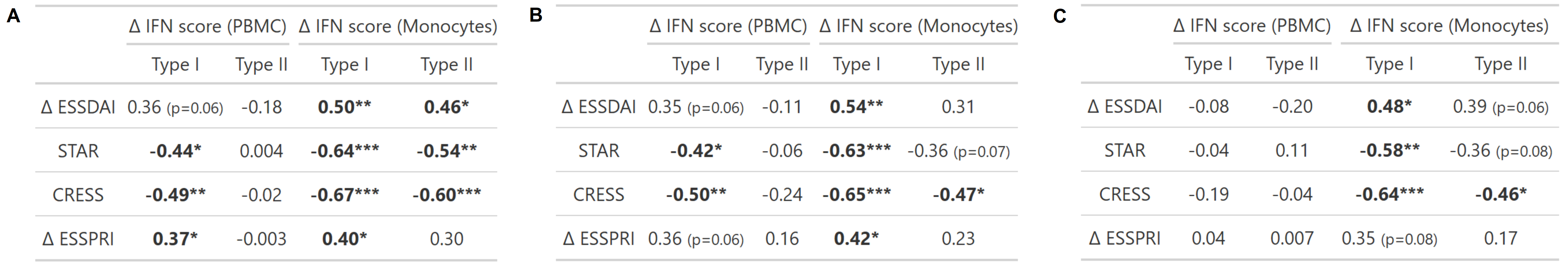

Supplement: Supplementary file 12 [file Image6.png]
